# Personally speaking: Developing and evaluating an ontology of dimensions of meaning for self-disclosure with a conversational assistant

**DOI:** 10.1371/journal.pone.0341640

**Published:** 2026-02-20

**Authors:** Libby Ferland, Hannah Qu, Wilma Koutstaal

**Affiliations:** 1 Department of Computer Science and Engineering, University of Minnesota, Minneapolis, Minnesota, United States of America,; 2 Department of Psychology, University of Minnesota, Minneapolis, Minnesota, United States of America; University of Mpumalanga, SOUTH AFRICA

## Abstract

Dialogue systems and conversational assistants are promising technologies given their general accessibility and appeal, but longer-term adoption often falters. Creating systems that engage users over the long term is a challenging design problem, largely because it depends on relationship formation between user and agent. Self-disclosure, or the act of revealing information about oneself, is a fundamental component of relationship building and maintenance between humans, and it has been shown to occur in interactions between humans and language-based systems as well. Disclosure on the part of users is an exceptionally rich source of information that has the potential to shape everything from user modeling to conversational experience design; however, that same richness makes interpreting disclosure difficult. Although some research has examined different sources of meaning such as topic and intimacy, the convergence of these sources of meaning under one umbrella has yet to be considered. We propose an ontology of self-disclosure with dialogue systems as a means to address this gap. The proposed ontology encapsulates previously explored dimensions of self-disclosure, such as topic and intimacy, as well as some additional novel layers of meaning, such as the separation of topic from the mental verb referred to in the disclosure (e.g., habit vs. preference vs. memory), in order to further discretize the separate dimensions of this complex phenomenon and make explicit potentially valuable sources of information for agents. We demonstrate an application of this ontology to instances of self-disclosure, drawn from real dialogues between users and a task-oriented conversational assistant, and examine the observed relationships between different dimensions of meaning. The practical implications of these findings, as well as the potential for further developing the ontology, demonstrate the usefulness and value of approaching self-disclosure as a multi-faceted, interconnected phenomenon.

## Introduction

The oft-noted tendency of humans to anthropomorphize – humanize – artefacts in their day-to-day lives can also be observed in interactions with technology. The human mind is tuned towards certain cues as indicators of a social presence – namely, language and voice-based interaction. The rise of conversational systems as technologies with immense potential in a number of applications means that the sociobehavioral aspects of interactions with conversational agents (CAs) cannot be overlooked, regardless of whether the CA is meant to be used in a task-oriented or social context. Prominent amongst the social behaviors that emerge as people navigate and negotiate relationships in their day-to-day lives is the phenomenon of self-disclosure. Known to be a core process in forming, deepening, and maintaining human-to-human relationships, users also self-disclose when interacting with CAs. From a human-computer interaction (HCI) perspective, self-disclosure carries with it both valuable opportunities and real risks – yet, to date, the computational and interaction design challenges it raises remain underexplored. This is, in large measure, for two reasons. First, most studies examining self-disclosure in computer-mediated contexts have been undertaken in highly socially-oriented contexts – that preclude exploration of the nature and occurrence of self-disclosure even when users are interacting with primarily task-oriented (minimally social, nonreciprocating) systems. Second, and more notably, many computational and interaction challenges arise because there is no unified ontology that captures the multiple discrete dimensions of meaning that may be present in even a single instance of self-disclosure.

Self-disclosure can be broadly defined as a voluntary process by which we share information about ourselves [1]. Self-disclosure occurs in a variety of circumstances, including human-human interactions, computer-mediated interactions [2–5], and interactions with intelligent systems (see e.g., [6–9]). In human-human interaction, self-disclosure is shown to be a primarily dyadic phenomenon driven by reciprocity between conversational partners [10, 11]. It is also often an iteratively deepening process as relationships become closer and more complex over time [10]. From a psychological perspective, self-disclosure is a nuanced, beneficial behavior. It can be an indicator of trust [12, 13], engender feelings of closeness when meeting new people [14], or increase intimacy between romantic partners [15, 16]. On a more intrapersonal level, self-disclosure can also be a form of stress management and catharsis, and serve as a pressure valve in times of distress [17, 18].

The growing body of evidence documenting self-disclosure as a commonly observed behavior in human-computer interaction suggests that similar processes and benefits may drive the use of self-disclosure in CA-based interactions as in human-human interaction. For example, people experience many of the same psychological benefits of self-disclosure with a conversational agent as they do in interactions with other humans [19]. There is also evidence that disclosing behaviors in human-agent interactions benefit from the same protective factors that may act to promote disclosing behaviors in computer-mediated communication, particularly

online. For example, the impression of anonymity may encourage more intimate disclosure faster [4, 20], and the impression that CAs are non-judgmental may further increase the speed at which disclosure becomes deeper [21–23].

These findings all point to a critical need for increased understanding of self-disclosure from a computational perspective as well as a psycholinguistic one. Reliably detecting and interpreting what is being disclosed by a user in CA interactions remains an open issue [24] – likely because fully understanding all of the sources of explicit and implicit meaning accompanying self-disclosure is a difficult problem from a machine perspective [9, 25]. The topic and the intimacy of disclosure, for example, are two sources of meaning that may exist both separately and in relation to one another; the content of self-disclosure is incredibly diverse and can encompass a variety of topics and emotional states, as well as have wildly varying levels of intimacy in what is disclosed (e.g., thoughts about body image and self may be much more intimate topics than personal preferences about food). Despite preliminary findings that a computational understanding of self-disclosure can be partially captured by examining such characteristics as its topic and level of intimacy, no unified model of the complexities and varied dimensions of self-disclosure currently exists. However, its importance as a tool in managing relationships with both humans and machines means that adopting a unified model such as is presented in this study may be beneficial to deepening understanding of the nuance of disclosure, and help develop an approach to tackling self-disclosure as a computational problem.

Self-disclosure, however, is also a design problem – particularly as the increasing societal relevance of conversational technology foregrounds long-standing issues in conversational interaction design. Dialogue systems may be used in comparatively unconstrained, open domain ways, as in the case of social support CAs or general question-answering agents, or be constrained to a primary set of tasks and use cases [26]. User interactions with both types of agents, however, are affected by the increasing power and apparent “humanness” of language models – potentially leading to a mismatch between users’ expectations and the reality of what CAs can actually do [27–31]. Powerful language models may exacerbate existing human tendencies to anthropomorphize intelligent agents and foster expectations of increasingly human-like behaviors that, when such behaviors are not forthcoming, can be detrimental to long-term adoption and user satisfaction [28, 29]. The ongoing questions of what a CA should understand, what it should respond to, and how it should do any of it are central to the community’s growing interest in addressing design issues that have thus far made long-term adoption of CAs one of the primary hurdles in their wider success (see, e.g., [27, 32–34], among others).

The expectation vs. experience gap is particularly challenging when it comes to systems designed with some sort of social capability in mind. Social presence, just like language, is an anthropomorphic cue, and an intelligent agent that creates an impression of a social presence may be especially prone to engendering the sort of anthropomorphization that can become problematic for users and user satisfaction [30, 35]. Many studies examining self-disclosure in human-agent contexts have employed CAs that create this impression and were explicitly designed to have some sort of social capabilities, including conveying the impression of reciprocity [7–9, 36]; though see also [5]). However, this emphasis on CAs with social features leaves entirely unexplored the key question of how and if self-disclosure may occur in more task-oriented systems – despite the myriad of potential uses for voice-driven but specifically task-focused intelligent agents for assisting in day-to-day activities. Fully modeling and analyzing self-disclosure from a computational (intelligent agent) perspective – in task-oriented systems – thus has considerable but thus far unrealized potential. Identifying, appropriately classifying, and contextually integrating what is revealed (directly or indirectly) during self-disclosing instances could be a rich source of information for enhancing and deepening user personalization, while side-stepping the problem of engendering expectations of reciprocity that cannot (over the longer-term) be met [25, 35].

The current two-phase study is designed to address questions about the presence of self-disclosure in human interactions with a task-oriented CA system assisting in day-to-day tasks. Specifically, we document the diverse variety of socio-emotional, conceptual, and other information that human-to-CA spoken self-disclosures can reveal, and explore how to meaningfully capture that informational richness in a unified ontology. In Phase I, we explore sociolinguistic characteristics of self-disclosure by analyzing participant conversations with a Wizard of Oz (WoZ) [37] ‘prototype’ task-oriented dialogue system designed to function as a daily planner. This phase was motivated by one overarching question:

**RQ1:** Do users of a primarily task-oriented WoZ system participate in self-disclosure? If so, how often do they do so, and what are the psycholinguistic characteristics of such disclosure?

In Phase II, we develop an ontology of different dimensions of meaning for self-disclosure and demonstrate its use in annotating instances of self-disclosure taken from the Phase I WoZ data. In this phase, we addressed 2 further research questions:

**RQ2:** Do instances of self-disclosure have a psycholinguistic profile distinct from the conversational turns around it? If so, what is the psycholinguistic profile of self-disclosure?
**RQ3:** How do the identified dimensions of meaning in the proposed ontology of self-disclosure relate to one another, given validated instances of self-disclosure as assessed by psycholinguistic analysis and human raters?

Our contributions in Phase I are as follows: 1) We show that users do fairly often participate in self-disclosing behaviors even with a primarily task-oriented CA system with minimum social capability, thereby showing that self-disclosure with CAs may not necessarily rely on reciprocity; and 2) We provide evidence that such self-disclosing behaviors continue across several sessions of interaction, suggesting that such self-disclosure is not exclusively driven by a novelty effect or by initial user exploration of the CA’s capabilities or functions. In Phase II our contributions include: 1) A proposed unified ontology capturing multiple discrete dimensions of meaning present in instances of self-disclosure; 2) An exploration of the psycholinguistic properties of the instances of self-disclosure found in Phase I as related to this ontology; and 3) An application of the proposed ontology by independent human raters to characterize instances of self-disclosure, and the potential extraction of direct and indirect inferences from those instances.

In this article we begin with an overview of related work, including an elaboration of the identified dimensions of meaning for self-disclosure that are used in our ontology. We then detail methods used in Phase I and discuss results from this phase as they pertain to **RQ1**. Next, we describe the methodology for development of the self-disclosure dataset used in Phase II, including the annotation of self-disclosure, and show the results of the annotation as well as analyses of the dimensions of self-disclosure that are central to **RQ2** and **RQ3**. We further outline the characterization of socially-grounded inferences about disclosers provided by human raters ( **RQ3**). We conclude with an exploration of real-world implications for an improved understanding of self-disclosure that occurs in dialogue with conversational systems, and a brief discussion of limitations and future directions suggested by this work.

## Related work

### Social interactions with technology

Anthropomorphization of and social interaction with computers and other technologies is a widely studied phenomenon. The Computers As Social Actors (CASA) paradigm is a well-supported conceptual framework with the core tenet that humans respond to technologies with social behaviors consistent with those characterizing human-human interactions [38, 39]. Work in the field so far has shown that this encompasses a wide variety of behaviors, primarily indicating that humans recognize technologies as a distinct social presence separate from themselves and generally demonstrate appropriate social responses to computer presences [38, 40]. For example, humans treat computers as a social source or actor [38, 41], differentiate between different systems as different social presences [38, 40], and even spontaneously interact with technologies using language that obeys social conventions such as politeness and friendliness [39, 42].

Work under the CASA paradigm further indicates that humans require minimum and rudimentary anthropomorphic and social cues to treat technologies as social actors (see e.g., [39, 41, 43–45], etc.). Multiple studies have demonstrated that humans are predisposed to social interaction and are prone to interpret cues in a social manner, which colors any interactions with technology [46]. Given this predisposition, individuals have been shown to respond more strongly than might otherwise be expected in view of the sparsity of social indicators actually provided [45].

From an HCI standpoint, several anthropomorphic/social cues stand out as key sources of this behavior: language-based technologies (either voice or text), interactivity over time, and the varied types of interactions a technology supports [41]. Given that human cognition is so well tuned to social interaction [45], minimal social cues may invoke a subconscious or ‘mindless’ social response [42, 45, 47] as the brain attempts to offload the amount of processing needed to interpret the environment and the assumed or implied presence of others. In this context, the CASA framework is particularly relevant to voice-activated and chatbot technologies, and plays a core role in relationship development with the same [48]. Further evidence also suggests these technologies are particularly prone to anthropomorphization due to their interactivity over the long term [41], which is especially relevant to systems expected to have developing and deepening relationships with humans as each interaction builds on previous ones [27, 48]. Nonetheless, the longer term trajectory of these modes of social interaction remain debated [32], with some researchers proposing a model in which long-term users accept CAs as a modified reduced anthropomorphic presence and do not expect full social behavior such as reciprocity in self-disclosure [48]. Others, however, propose that self-disclosure, especially reciprocal disclosure, is an important part of building a relationship with a conversational system, as well as user satisfaction in long-term use [5, 7].

### Self-disclosure

Self-disclosure, or the voluntary act of sharing information about oneself, is a fundamental part of human-human interaction and communication [1, 10, 11]. The act of disclosing is primarily a relationship formation and maintenance activity, and is one of the keys to building positive and more intimate relationships of all kinds (see, e.g., [10, 11, 15, 16]). In human-human interactions, several defining characteristics of self-disclosure have emerged: it is a dyadic process, both parties in a dyad participate in approximately the same level of disclosure, and disclosure is a reciprocal process that deepens over time [6, 10, 11]. Reciprocity is thought to be one of the most important parts of self-disclosure as a social process and one of the core components of developing more intimate and lasting relationships; thus, here we use Collins & Miller’s [10] definition of reciprocity in self-disclosure as a conversational partner responding to self-disclosure with self-disclosure of equal intimacy. Therefore, a non-reciprocating partner is one who either does not disclose or provides some minimal acknowledgment that disclosure has occurred, without engaging with what has been disclosed [5].

Due to its nature as a fundamental part of the relationship building process, self-disclosure is a phenomenon also demonstrated through the CASA framework [19]. Therefore, it stands to reason that agent and interaction designs should account for the possibility of user self-disclosure as an important tool in building rapport and increasing user acceptance [6, 49]. There is an increasing body of evidence that users may experience many positive effects of interacting with conversational agents, sometimes even markedly more so than in face-to-face interactions with humans [19, 50]. In fact, the previously discussed benefits of self-disclosure appear to exist in human-agent interactions just as they do in human-human interaction [19], and evidence suggests that certain qualities of agents such as their being nonjudgmental may actually increase a human user’s willingness to disclose [21]. The type and amount of self-disclosure a user participates in during the course of an interaction can be affected by a number of factors, many of which can be adjusted in the course of initial agent design or can be dynamically and responsively modified during the ongoing interaction by the agent itself.

### Dimensions of meaning in self-disclosure

While the act of self-disclosing has many different social aspects, the self-disclosure itself can also be analyzed using multiple dimensions. Previous research in computer-mediated conversation and in interactions with intelligent agents has considered a number of dimensions, such as the topics being discussed in the disclosure, the implied intimacy of what is being disclosed, and the emotional valence (positive or negative) of disclosure statements. The six-dimension ontology proposed in this work expands on the previous understanding of dimensions of self-disclosure with the addition of several important dimensions of meaning: the underlying cognitive or psychological processes that are referred to or implicated by the disclosure (mental verb), the context of the disclosure in conversation, and what inferences about the discloser, if any, might be drawn from the disclosure.

The identification of these dimensions not only makes explicit the ways in which humans interpret disclosure statements, but importantly also identifies dimensions of meaning that may assist in training intelligent agents to appropriately interpret and interact with self-disclosure in a conversational setting. One of the key potential uses for this kind of “interpreting between the lines” from a machine perspective is developing a better knowledge base for users (more robust user modeling), as well as facilitating more appropriate interactions with user information in dialogue generation [9, 25]. Thus, assessing and characterizing some of these dimensions (e.g., the context of the disclosure and the implications of what is disclosed) may be especially central to developing and implementing improved machine understanding, though more thoroughly and precisely characterizing these aspects may also contribute to our psychological understanding of human-to-human and other computer-mediated interactions.

The three novel dimensions present in our ontology are particularly salient in developing this deeper understanding because they capture what was previously left implicit or intermingled with other dimensions; for instance, the pioneering questionnaire developed by Jourard & Lasakow makes no differentiation between the content/topic of self-disclosure and its underlying mental verb [1]. Understanding the underlying psychological processes behind different types of self-disclosure – the mental verb – as well as gaining more detailed knowledge of the conversational contexts in which self-disclosure occurs may help in developing more complete user-specific profiles to further tailor conversations and conversational style from a CA perspective. The implications, or inferences, behind an instance of self-disclosure are also an important novel aspect from a knowledge organization perspective and can assist in determining what sort of background knowledge or reasoning capabilities are necessary for a system to detect, interpret, and properly respond to information being disclosed. The six dimensions of self-disclosure we examine here – *topic, mental verb, intimacy, emotional valence, conversational context, and inference/implication* – taken both separately and together, can help us gain a more complete understanding of the sources of meaning in self-disclosure in conversational interactions. We attempt to isolate and capture these nuanced dimensions of meaning in the hopes of unifying previously disparate computational problems into a comprehensive framework, and further enriching the feature space of future data used in training intelligent systems.

Here we further describe each dimension included in the proposed ontology of self-disclosure. We present the dimensions in the same order in which they were presented to human raters (annotators) in Phase II of the study. This means that the three novel dimensions described are interleaved with the other dimensions. This was done (for raters and readers) both to highlight some potential relationships between dimensions and to emphasize that, while related, these dimensions are distinct. Table 1 provides an overview of the six identified dimensions of meaning.

**Table 1:** Summary of dimensions of self-disclosure.

Dimension	Definition
Topic	What knowledge base category the disclosure belongs to (leisure, health, finances, etc.).
Mental verb	The mental activity referred to in the disclosure (e.g. memory, preference).
Emotional valence	The positivity or negativity of the disclosure.
Intimacy	How personal or sensitive the disclosure is.
Conversational context	The degree to which what is disclosed follows or deviates from prior conversational context (e.g., relatedness to prior turns).
Inference	What additional information might be inferred about the discloser.

Each dimension identified in this ontology provides an important piece of context critical for fully interpreting and interacting with self-disclosure. From content domain (topic, mental verb) to socioemotional context (emotional valence, intimacy) to conversational continuity and relational context, each dimension captures a distinct and yet interrelated facet of self-disclosure in conversation. These dimensions include:

**Topic.** One of the hallmark forays into self-disclosure research came from Jourard and Lasakow’s work in the development of a self-disclosure questionnaire (the Jourard Self-Disclosure Questionnaire, or JDSQ) [1]. The questionnaire identified 6 major categories of self-disclosure that people may participate in: attitudes and opinions (beliefs), tastes and interests (preferences), work or studies, money or finances, personality and self, and body. These broad categories are often still used today to define potential topics of self-disclosure (e.g., [3, 4]). The breadth of topics discussed is also a defining characteristic of self-disclosure [51, 52], so considering potential topics being discussed is important in characterizing self-disclosure with a conversational agent. From the perspective of agent design and development, topic modeling and detection may help with knowledge organization and contribute to appropriately interpreting the content of self-disclosure [53].

**Mental verb.** Beyond the content of self-disclosure, the mental verb of self-disclosure has been seldom investigated as its own separate dimension. The topics traditionally investigated in self-disclosure (e.g., leisure, finances, friendship) are the *nouns* of the act – but just as in linguistics, the mental *verb* or mental activity referenced in self-disclosure plays an important role in how topics relate to one another [54, 55]. These mental verbs are related to folk psychology theories of mind, and represent the common-sense understandings of the mind that people tend to use in day-to-day life [56–58]. These verbs represent distinct aspects of sensing and interacting with the world and the self, and are important parts of the mental models people maintain for themselves and for others they interact with socially [59–61]. The psychological states referred to by mental verbs are considered distinct from their content. Goldman (1993) most succinctly described this idea in a discussion of mental attitudes: “Wanting there to be peace and believing there will be peace are different attitudes because their types (wanting and believing) are different. Intending to go shopping and intending to go home are different attitudes because, although their type is the same, their contents differ [62].”

D’Andrade (1987) suggests that mental verbs (in the English language) can be classified into six types: perceptions (e.g., see, hear), belief/knowledge (e.g., believe, remember), feelings/emotions (e.g., approve, feel happy), desires/wishes (e.g., want to, hope to), intentions (e.g., plan to, aim to), and resolutions (e.g., determined to, resolve to) [56]. These distinct verb types parallel the mental verbs in the proposed ontology. Table 2 demonstrates the expression of the same topic in many different ways using our ontological categories. Each of these expressions represents or refers to a distinct mental state on the part of the speaker, and understanding how to interpret that mental state is important in ongoing interactions. A savvy listener might use the perceived mental state as a touchstone for further conversation, whether it be in selecting a properly empathetic response, eliciting further follow-up dialogue, or more proactively guiding a conversation in a particular direction. Agents able to detect underlying mental verbs may therefore have an advantage when it comes to appropriately responding to user disclosure, which is an important aspect of building and maintaining social relationships [63]. Another layer of complexity is that agents may need to be trained not only to identify psychological states that are explicitly indicated in a user’s conversation by a mental verb (e.g., “I believe,” “I prefer”) but also psychological states expressed indirectly (e.g., “It is my opinion that x,” “I have a strong preference for x”). In these examples, “x” refers to the topic, but “my opinion” and “preference” refer to psychological states/mental verbs.

**Table 2:** Mental verbs.

Disclosure	Mental Verb
I like short ribs so much better than sirloin when I make beef stew.	Preference
Seeing people enjoy my beef stew makes me so happy.	Feeling/emotional state
The best markdowns on stew beef seem to happen on Mondays.	Thought/belief or judgement
I make beef stew once a week.	Habit
I’m going to pick up some short ribs for stew today after work.	Planning
I burned my beef stew once, it was the only time I’ve ever used a fire extinguisher.	Memory
The butcher I buy beef from marks sirloin as stew beef.	Factual
My beef stew, beef stew, whatcha gonna do?	Other

Statements on the same topic (beef stew) expressed through different mental verbs.

**Intimacy.** The intimacy of self-disclosure refers to how public or private (personal) the items being discussed might be. This dimension is also heavily linked to the topic of conversation and the broader environmental or social contexts in which a conversation is taking place. Previous work has found strong positive correlations between different topics of discussion and the perceived intimacy of the conversation [3, 4]. More recent work has found that the same general patterns between topic and perceived levels of intimacy also apply in interactions with CAs. This may be particularly impactful for interactions with agents, since the types of topics a user is willing to discuss with a conversational system may be affected by environmental/social contextual aspects such as a dialogue system’s placement or location within a home [48, 64].

**Emotional valence.** The valence of self-disclosure refers to the emotional content of the disclosure – in particular, how positive or negative the self-disclosure is from an affective/emotional perspective. Evidence suggests that while users may have preferences over what topics to discuss/disclose with an agent vs. a human, agents such as chatbots may elicit more negatively valenced instances of self-disclosure than would occur with a human listener [22]. There is also growing evidence that valence of disclosure is at the very least related to the topics of disclosure [65, 66]. However, the relationship of valence to other measurable aspects of self-disclosure is an open question. From a computational (agent processing) perspective, accurately measuring valence assists in responding to self-disclosure with appropriate empathy or in an otherwise contextually appropriate way, which is an important tool in human-agent relationship building [26, 63, 67].

**Conversational context.** Conversational context is a dimension that may be especially relevant to machine understanding of self-disclosure. From a computational perspective, the relatedness of topics between speaker turns may give insight as to how much conversational history needs to be maintained in order to properly interpret self-disclosure, and may also impact knowledge organization. To evaluate the relatedness of topics, we gathered ratings of the relevance of the self-disclosure statement to the immediately previous statement/prompt by the CA in the conversation. Self-disclosure by definition does not occur in a vacuum; however, there may be instances in which the logical leap from a CA dialogue turn/prompt to what is being disclosed by a user is unclear, particularly from a machine perspective. We attempt to capture this dimension of meaning to better understand the roles background knowledge and particularly an awareness of conversational history play in appropriately understanding instances of self-disclosure.

**Rater-provided inferences.** Detecting the deeper meanings that can be drawn from instances of self-disclosure is also an open question. Humans may infer information about a speaker from what is disclosed that an intelligent agent may not be able to interpret. Examining the inferences human raters make when judging self-disclosure allows us to further narrow down what is being disclosed and what background or social knowledge is necessary in order to interact with self-disclosure in a human-like fashion. This is a key dimension from a machine perspective because identifying the background knowledge that is necessary to understand conversations is still limited, greatly restricting the social reasoning abilities of agents [68]. Increasing social and emotional understanding by “reading between the lines” also contributes to building and maintaining more productive relationships with CAs [67].

**Fig 1:**
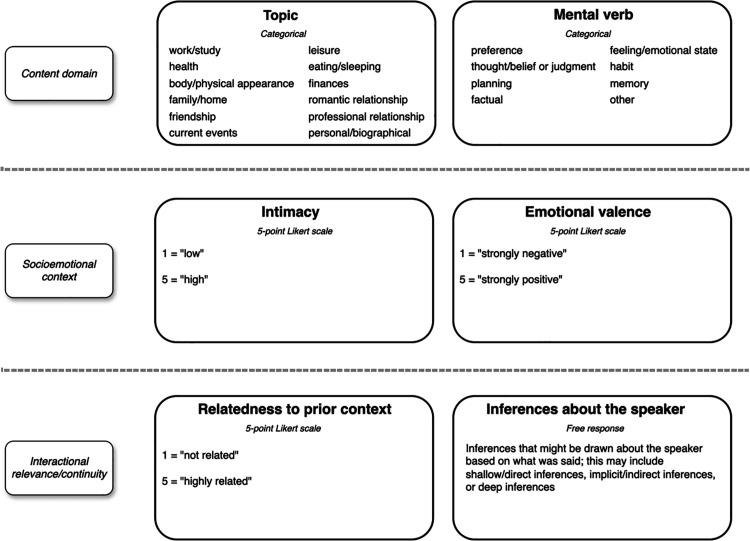
The six-dimension proposed ontology of self-disclosure. The proposed ontology illustrates that self-disclosure is a highly nuanced process, and interpreting it requires socioemotional, situational, and personal context.

Taken together, these six dimensions capture multiple aspects of the complex phenomenon that is self-disclosure. Fig 1 demonstrates the ontology, including scales and categories used for each dimension.

## Experimental overview

**Fig 2:**
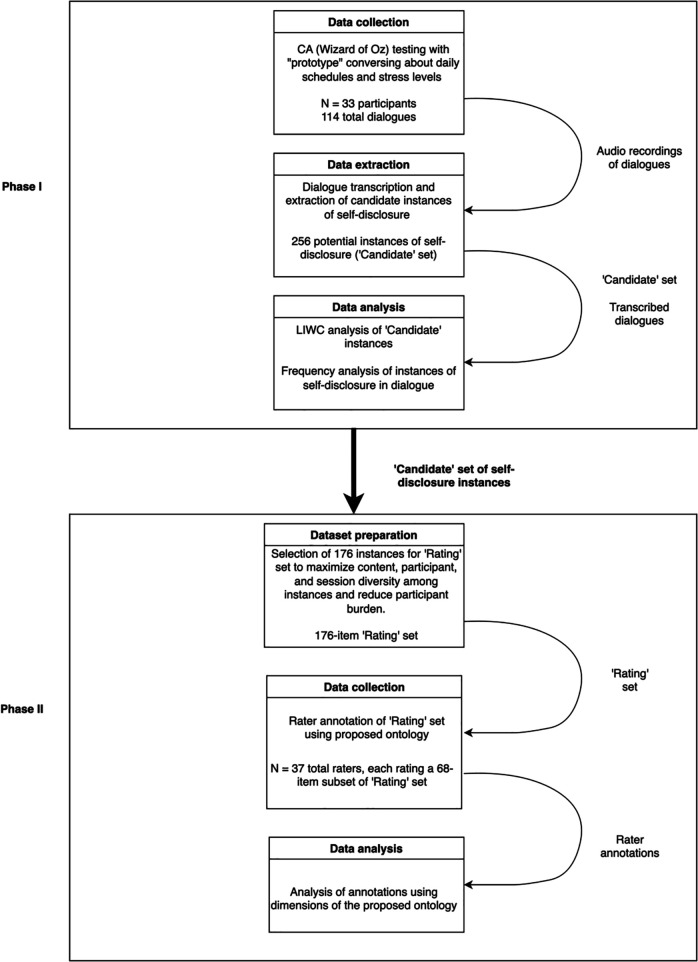
Full experimental overview for Phases I and II. A demonstration of the workflow and data flow for Phases I and II. Each phase involved different groups of participants; one group (Phase I) generated conversational data, and one group (Phase II) evaluated transcripts of those conversations.

This study consisted of two separate phases, covering both conversational data collection and the development and application of the self-disclosure ontology. Each phase had a different group of participants. In Phase I, the first group of participants generated conversational data by interacting with a ‘prototype’ conversational agent about daily scheduling and giving numerical ratings of event-related stress. In Phase II, the second group of participants evaluated transcripts of these conversations using the proposed six-dimension ontology of self-disclosure. In this article, we describe each phase sequentially, including an overview of the methodology and protocols for each experiment and analysis of the data gathered in each phase. Fig 2 provides an overview of the workflow for the entire study, including both Phases I and II.

## Phase I: Conversational data collection

Phase I of this study involved a WoZ study from which transcripts were collected and analyzed. The primary aim of Phase I was to examine **RQ1** and its subquestions – namely, does self-disclosure occur when users interact with a task-based dialogue agent, and if it does, what are the general characteristics of those interactions from a psycholinguistic standpoint?

### Methods and tools

#### Ethics statement.

This research (both Phase I and Phase II) was conducted in accordance with the approved IRB protocol from the University of Minnesota (STUDY00004825), “Tell Me About Your Day: Evaluating Naive User Interactions with Smart Home Assistants.” Written informed consent was obtained from all participants in Phase I, including consent to analyze and publish anonymized transcripts of their interactions with the WoZ system. Participants were informed of the use of deception as part of the WoZ protocol, and were given an additional opportunity to modify or withdraw consent after deception was disclosed.

#### Psycholinguistic analysis and linguistic inquiry word count.

Linguistic Inquiry Word Count (LIWC) provides both simple counts of the frequency of words in different semantic and syntactic categories, and standardized measures assessing particular semantic and sociolinguistic properties [69]. We focused on four summary variables (standardized measures based on a 100-point scale ranging from 0 to 100) to characterize the language usage of participants with the CA [70]:

(1) Analytical thinking. A high score means a more formal and logical response, and a low score means a less structured or less reserved response. Participants getting a lower score in Analytical thinking suggests that they may feel more comfortable or relaxed with the CA.
(2) Clout. A high score means a more authoritative and confident response, and a low score means a more humble and tentative tone.
(3) Authenticity. A higher score means a more self-disclosing response, and a lower score means a more guarded response.
(4) Emotional tone. A high score means a more positive tone, and a low score indicates anxiety or hostility.

We also considered a few other raw-word-count variables such as frequency of use of the personal pronoun “I”, words relating to participants’ cognitive processes (e.g., expressions relating to tentativeness or insight), and social-related words (e.g., referring to family or friends). A higher frequency of these variables may indicate more self-disclosure [70].

#### WoZ system design.

The dialogue system used in this data collection was a WoZ “prototype” [37] controlled by a member of the research team. The research team attempted to give as strong an impression of realism as possible in both the experimental design and in creating the appearance of the WoZ system. To maximize verisimilitude, a researcher controlled the system from a separate room entirely hidden from participants, and participants spoke to a “prototype” consisting of a Raspberry Pi board connected to a Bluetooth speaker. The system, which was given the working name ‘Joanna,’ was operated via a custom-designed soundboard with a very formulaic conversational design. ‘Joanna’ acted primarily as a verbal scheduling assistant for both prospective and retrospective uses. In the prospective case, users were prompted for information about sequential items on their schedule for the day, including start and end time, location, a name for the schedule item, any reminders about the schedule item, and a projected numerical rating of how much stress the user believes they will experience at that time. In the retrospective case, users were asked to give a numerical rating corresponding to how much stress they *actually* experienced during the schedule item. ‘Joanna’ was intentionally designed to minimize social cues as defined by Feine *et al*.’s taxonomy [71]; the ‘agent’ used minimal self-referential language during interactions, and did not offer opinions or advice, tell jokes, or otherwise affect a persona. Intentional social cues were limited to brief greetings (including ‘good afternoon’ or ‘good morning’ and ‘goodbye’), thanking, and a single ‘how are you doing today?’ small talk question at the beginning of the interaction with a static ‘I am doing well, thank you,’ response for instances in which a user reciprocated the question. In the retrospective case there was an additional ‘how did it go?’ social nicety question when prompting for discussion of past events. Most notably in this design was the absence of any self-disclosure on the part of the agent, meaning that the system was fully non-reciprocal to any instances of user self-disclosure and therefore intentionally did not give one of the primary cues that might increase the chance of ongoing self-disclosure in interactions.

### Experimental design

#### Experimental sessions.

Participants attended a total of 4 sessions spread over two days, and interacted with a personal digital assistant “prototype” in each session. Participants were asked to prospectively and retrospectively discuss events on their schedule for the day, including habits and items such as commutes and meals that may not typically appear on a calendar. Participants were further asked to rate their stress levels corresponding to each event on a scale from one to seven as well as their current mood and stress level. Physiological measures of participant stress (both during and between sessions) were also obtained, but are discussed separately [72]. This study was performed with IRB approval, including the use of deception, and participants were informed of the use of deception at the end of the fourth and final session. Participants received monetary compensation as well as course credit, administered through a University-approved system, for psychology courses which none of the researchers were involved with.

After the disclosure of the use of deception, participants were informally debriefed regarding the verisimilitude of the “prototype,” including whether or not they suspected they were interacting with a human rather than a machine; if they suspected they were interacting with a human, they were also asked to indicate when this suspicion occurred during the course of the experiment.

#### Data processing.

Following data collection, each experimental session was transcribed by the research team. Once transcription was complete, four members of the research team then reviewed each transcript to manually identify all potential instances of self-disclosure. In Phase I, the research team identified 256 candidate instances of self-disclosure over all participants and experimental sessions (‘Candidates’ dataset).

### Participant demographics

Recruitment and data collection for Phase I participants ran from 27 February, 2019 until 7 May, 2020. The total N for all participants in Phase I was 33, covering a total of 114 interactions with the CA. All participants were 18 years old or older, self-reported as having normal or corrected-to-normal hearing, and as having begun speaking English before 6 years of age; 22 were female, 9 were male, and 2 declined to identify.

### Results

**RQ1** was whether or not disclosing behaviors would be seen in user interactions with a task-oriented system. The answer to **RQ1** is uncategorically *yes*. Across all 114 CA-participant interaction sessions, there were a total of 256 dialogue pairs identified as candidate instances of self-disclosure. Out of 33 participants, 28 showed one or more candidate instances of self-disclosure, though the number of candidates amongst those 28 participants varied widely per participant (minimum = 1, maximum = 37, M = 9.14, Mdn = 5.50, SD = 8.92). For Phase I data, please see https://dataverse.harvard.edu/dataverse/personally_speaking [73].

As part of addressing **RQ1**, we investigated the psycholinguistic characteristics of the dialogues that users had with the CA, and how those characteristics interrelated with the amount of self-disclosure that occurred. LIWC analyses of the full context of participants’ conversations – that is, their full dialogue with the CA across the sessions – revealed several key trends among both the statistical summary variables and some of the dictionary-based raw word count variables. These trends are summarized in Table 3.

**Table 3:** Correlation of frequency of self-disclosure and LIWC characteristics in dialogues.

LIWC variable	Frequency of self-disclosure
Word count	r = 0.45, p = 0.017
Authenticity	r = 0.50, p = 0.006
Analytical language	r = -0.41, p = 0.031
Use of “I”	r = 0.54, p = 0.003
References to cognitive processes	r = 0.28, p = 0.045
Positive affect-related language	r = -0.44, p = 0.021

**Fig 3:**
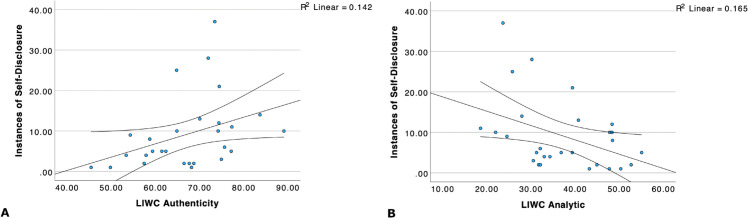
LIWC measures of authenticity and analytical language vs. frequency of self-disclosure. A: The LIWC authenticity measure is significantly positively correlated with frequency of self-disclosure. B: The LIWC analytical measure is significantly negatively correlated to frequency of self-disclosure. Note the values on the x axes differ.

First, among the LIWC summary variables (Word count, Clout, Authenticity, Analytical language, Emotional tone), the number of self-disclosures was positively correlated with Word count and Authenticity, and negatively correlated with Analytical language. These summary variables represent the highest-level trends in dialogue; thus, a visual representation of these trends using scatterplots corresponding to the latter two correlations are provided in Fig 3.

Additionally, as can also be seen in Table 3, several of the dictionary-based LIWC variables demonstrated interesting relationships with the frequency of self-disclosure. The frequency of using the personal pronoun “I” was significantly positively correlated with the number of instances of self-disclosure. This is consistent with the positive association of the frequency of self-disclosure with Authenticity. References to cognitive processes also tended to be positively related to self-disclosure, consistent with both the positive association of frequency and Authenticity and the negative association of frequency with Analytical language. In contrast, positive affect-related language was negatively associated with the number of instances of self-disclosure – indicating that participants with a higher overall level of positively-valenced language were less likely to engage in self-disclosure.

Were these instances of self-disclosure perhaps attributable to the CA involving a Wizard of Oz set-up? We performed some exploratory analyses to examine if users’ beliefs about whether or not they were speaking to a human had any effect on disclosing behavior. Informal debriefing of participants suggested that our carefully devised two-room experimental set-up aiming for high verisimilitude to a prototype CA was largely successful, in that the majority of participants remained naïve to the Wizard of Oz set-up and post-hoc – even after being debriefed – reported only a few specific interactional exchanges, across the four sessions, that had prompted them to wonder if the CA was perhaps not fully automated and potentially under human guidance. There were 11 participants who indicated that they had never – at any time during the four different experimental sessions – suspected that a human might be aiding the CA. These entirely naïve participants had a mean number of 9.45 instances of self-disclosure (Mdn = 4.00), thus reflecting an average rate of self-disclosure closely mirroring that shown by the full sample (M = 9.14, Mdn = 5.50). Notably, the frequency of self-disclosure among the entirely naïve participants significantly exceeds what might be anticipated if humans engaged in no self-disclosure with a task-oriented and minimally socially-reciprocating CA (one-sample t-test against zero, t(10) = 3.06, p = .012, Cohen’s d = .92, 95% CI [.19, 1.62]).

## Phase II: The ontology

In Phase II of the study, we recruited independent human raters to evaluate transcriptions of the conversational data from Phase I. Using dialogue snippets, raters were asked to apply the proposed six-dimension ontology to each. In Phase II, we used analyses of these ratings to address **RQ2** (how do we differentiate instances of self-disclosure from surrounding text?) and **RQ3** (how do the dimensions of self-disclosure in the proposed ontology relate to one another?).

### Methods and tools

#### Ethics statement.

This research (both Phase I and Phase II) was conducted in accordance with the approved IRB protocol from the University of Minnesota (STUDY00004825), “Tell Me About Your Day: Evaluating Naive User Interactions with Smart Home Assistants.” Written informed consent was obtained from all participants in Phase II.

#### Self-disclosure survey.

**Fig 4:**
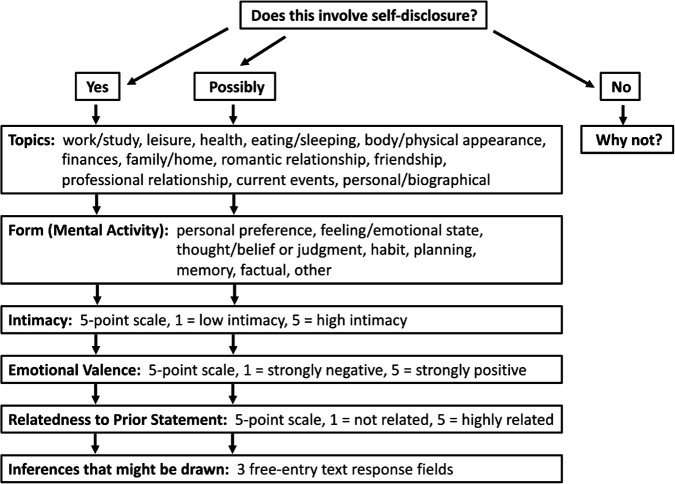
Workflow for Phase II patings. Participant workflow in evaluating stimulus pairs, including the seven rating items.

We developed a survey asking participants to evaluate six different dimensions of meaning in instances of self-disclosure. The survey was designed to both align ourselves with existing literature and to address research questions of interest. Questions concerning the six different dimensions of meaning were preceded by an over-arching question as to whether self-disclosure occurred in a given utterance. Altogether, the following seven items (diagrammed in Fig 4) were evaluated in the survey:

(1) Whether or not a sample contained self-disclosure. Raters were given the options ‘yes’, ‘possibly’, or ‘no.’ Raters who selected ‘no’ were further asked to justify their choice as a manipulation/attention check.
(2) The topic being discussed in the self-disclosure (content). Raters were asked to select their top two or three descriptions of the topics being discussed. There were 12 topics drawn and subdivided from the initial categories in the Jourard Self-Disclosure Questionnaire (JSDQ) [1], plus an ‘other’ category. The 12 JSDQ-based topics included work/study, leisure, health, eating/sleeping, body/physical appearance, finances, family/home, romantic relationship, friendship, professional relationship, current events, and personal/biographical.
(3) The way in which the disclosure was expressed (mental verb). Raters were again asked to select their top two or three choices regarding the mental verb referred to in the disclosure. There were 8 mental verb categories, including preference, feeling/emotional state, thought/belief or judgment, habit, planning, memory, factual, and other.
(4) The intimacy level of the disclosure. Raters were asked to evaluate the sample using a five-point Likert scale anchored at 1 for low and 5 for high intimacy.
(5) The valence of the disclosure. Raters were asked to evaluate the sample using a five-point Likert scale anchored at 1 for strongly negative and 5 for strongly positive emotional valence.
(6) The relatedness of the disclosure to the previous conversational context. Raters were asked to evaluate the sample using a five-point Likert scale anchored at 1 for not related and 5 for highly related to the previous statement by the first speaker.
(7) The inferences that can be drawn about the speaker from what was disclosed. Examples were provided. Raters were given three free-entry response options to indicate any information they might infer about the speaker from what was disclosed. Raters were also told that they could include restatements of the information contained in the disclosure if they had difficulty drawing inferences.

The evaluation process for each stimulus pair is illustrated in Fig 4.

### Experimental design

#### Data structuring.

Following the selection of the 256-item ‘Candidate’ self-disclosure dataset, a team of expert raters then identified a narrower subset of 176 agreed-upon potential instances of self-disclosure (‘Rating’ set). The items in the ‘Rating’ set were chosen to represent as many different participants and experimental sessions as possible, while also sampling a wide range of content. Each instance of self-disclosure (“stimulus pair”) was a pair of utterances consisting of a statement/prompt from the CA (listed as Speaker 1) followed by a response from the Phase I participant (listed as Speaker 2).

#### Experimental protocol.

The identified self-disclosure stimulus pairs were divided into 5 sets. One set consisted of a representative sampling of 32 of the stimulus pairs that all participants were asked to rate (the “All” or H set). The remaining stimulus pairs were distributed into four sets of 36 items each. To reduce participant burden, subsets of participants were randomly assigned to rate one of these four subsets (Set A: N = 9, Set B: N = 9, Set C: N = 11, Set D: N = 8). Each participant therefore rated 68 stimulus pairs (32  +  36) using the 7-item rating scheme. The stimulus sets were subdivided and interleaved, so each participant viewed one of four different orderings. For example, a participant may have rated 8 items from the H set, 9 items from the A set, 8 items from the H set, and so on. This was done to alleviate temporal effects.

### Participant demographics

Recruitment and data collection for Phase II participants ran from 14 June, 2021 to 13 August, 2021. The total N for all participants was 37. All participants were aged 18 or older; ages ranged from 18 to 55 with a mean age of 22.4 years and a median age of 20. All participants identified as native English speakers (started speaking English before 6 years of age); 29 participants identified as female, 7 as male, and 1 as non-binary.

### Results

Our analysis is based primarily on transcriptions of the user interactions with the CA that occurred in Phase I. We focus on transcript data as a behavioral measure as the CA in this protocol was a unimodal voice-activated system. Additional analysis is based upon the ratings of self-disclosure completed by participants in Phase II. For Phase II data, please see https://dataverse.harvard.edu/dataverse/personally_speaking [73].

#### Psycholinguistic profile of self-disclosure in context.

In order to further verify our rating scheme for self-disclosure, as well as the accuracy of human raters in performing the task, we addressed **RQ2** by analyzing the psycholinguistic characteristics of rater-agreed-upon instances of self-disclosure using LIWC. The trends present in these LIWC analyses may both reflect – and support – dimensions of self-disclosure observed in previous research.

We used two types of control stimuli for this analysis, referred to as “control before” and “control after.” Specifically, for each of the 176 possible instances of self-disclosure in the Rating set, we returned to the relevant full transcription of that participant’s interaction session with the CA. We then extracted two groups of dialogue text: the words spoken by that participant to the CA at two dialogue turns before the identified instance, and the words spoken by that participant to the CA at two dialogue turns after the identified instance. If a given instance of possible self-disclosure occurred either very early or very late in the participant’s interaction session with the CA, then no control text could be extracted; additionally, if the text at the designated juncture was itself also among the other previously identified instances of possible self-disclosure, then no control text was extracted for that case. Through this process we obtained a total of 163 “control before” and 163 “control after” dialogue stimuli.

We then contrasted LIWC sociolinguistic characteristics for these control dialogue stimuli with four subsets of the Phase II rated instances of self-disclosure. Two of these subsets were based on rater responses to the first item in the ontology, which was whether or not a given dialogue snippet contained self-disclosure. Additional subsets were based on rater evaluations of the dimension of intimacy on a 5-point Likert scale:

1. Instances that 85% or more of participants rated ‘yes’ or ‘possibly,’ termed ‘high agreement is SD’ (*n*_*stimuli*_ = 87)
2. Instances that 66% to 84% of participants rated ‘yes’ or ‘possibly,’ termed ‘moderate agreement is SD’ (*n*_*stimuli*_ = 49)
3. Instances with a mean intimacy of self-disclosure rating greater than 3.10, termed ‘high intimacy SD’ (*n*_*stimuli*_ = 30)
4. Instances with a mean rated intimacy of self-disclosure lower than 2.90, ‘low intimacy SD’ (*n*_*stimuli*_ = 124)

**Fig 5:**
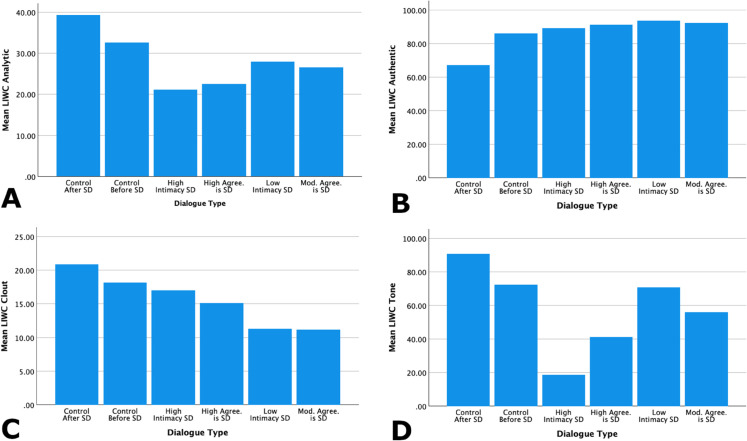
Linguistic profiles of self-disclosure in conversational context: LIWC summary measures. Trends among the four LIWC summary measures (analytical language, authenticity, clout, and emotional tone) in relation to preceding and succeeding dialogue turns. “Control before” and “control after” utterances are included in the leftmost two bars, high self-disclosure utterances are in the middle two bars, and low to moderate self-disclosure are in the rightmost two bars. A: Analytical language. B: Authenticity. C: Clout. D: Tone. Note: Values on the y axes differ between plots. SD = self-disclosure, Mod. = Moderate, Agree. = Extent of across-rater agreement in classifying the dialogue instance as involving self-disclosure.

The four LIWC summary variables show interesting trends in the language of self-disclosure relative to the surrounding conversational context. As demonstrated in Table 4 and Fig 5 we can see that, compared with both types of control dialogues (Control After and Control Before), the instances involving self-disclosure are perhaps qualitatively characterized by somewhat lower Analytical language and lower positive tone. Positive tone seems especially lower for instances judged as involving a high level of intimacy and that elicited high rates of agreement as self-disclosure. For the measure of Authenticity, there do not appear to be marked qualitative differences across the dialogue types except perhaps a decrease in Authenticity (from generally consistently quite high levels) for the “Control After” dialogues. Additionally, although the overall levels of Clout are generally low, Clout appears perhaps lowest for instances judged as involving a low degree of self-disclosure and that elicited moderate rates of classification as self-disclosure.

**Table 4:** Linguistic characteristics of self-disclosure in ongoing conversational context: LIWC summary variables.

LIWC Variable	Self-Disclosure Dialogue				Control Dialogue	
	H. agree.	Mod. agree.	H. intim.	L. intim.	Before	After
Analytical	22.56	26.54	21.16	27.93	32.59	39.39
Authenticity	91.29	92.39	89.20	93.63	86.10	67.21
(Positive) Tone	41.13	56.01	18.45	70.81	72.32	90.69
Clout	15.14	11.17	16.99	11.29	18.18	20.89

Note: The control dialogue is based on the within-person ongoing interaction, including the dialogue turns just “before” and immediately “after” each instance of self-disclosure. H. = High, Mod. = Moderate, L. = Low, Agree. = Extent of across-rater agreement in classifying the dialogue instance as involving self-disclosure. Intim. = Rater assessment of the level of intimacy in the dialogue instance.

We further analyzed several other LIWC variables to create a more detailed linguistic profile of self-disclosure. This analysis indicated important trends in instances of self-disclosure, namely in the use of personal pronouns (“I”), cognitive processes, social references, motivational drives, and positive and negative affect.

**Fig 6:**
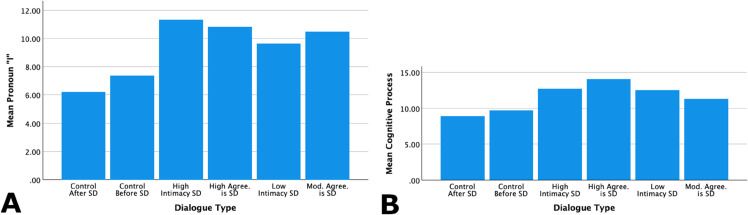
Further linguistic profiles of self-disclosure in conversational context: “I” and cognitive processes. “Control before” and “control after” utterances are included in the leftmost two bars, high self-disclosure utterances are in the middle two bars, and low to moderate self-disclosure are in the rightmost two bars. A: Use of “I” is highest in instances of self-disclosure compared to control dialogue. B: Language related to cognitive processes is higher in instances of self-disclosure compared to control dialogue. Note: Values on the y axes differ between plots. SD = self-disclosure, Mod. = Moderate, Agree. = Extent of across-rater agreement in classifying the dialogue instance as involving self-disclosure.

**Personal pronoun “I” and cognitive processes.** The use of the personal pronoun “I” was, as anticipated, more frequent during participants’ dialogue with the CA that was judged to involve self-disclosure than in the two types of control dialogue. A similar pattern was observed for the frequency of words referring to cognitive processes. Words in this category represent specific types of cognition, such as causal thinking (“because,” “why,” “how,” etc.) or insight (“know,” “think,” “feel,” etc.) [69]. The use of cognitive process words was elevated in dialogue judged as containing self-disclosure compared to control dialogue. These analyses are demonstrated in Table 5 and Fig 6.

**Table 5:** Linguistic characteristics of self-disclosure in ongoing conversational context: Personal pronouns (“I”) and cognitive processes.

LIWC Variable	Self-Disclosure Dialogue				Control Dialogue	
	H. agree.	Mod. agree.	H. intim.	L. intim.	Before	After
Personal pronouns	10.82	10.47	11.33	9.63	7.37	6.23
Cognitive processes	14.04	11.32	12.72	12.56	9.68	8.93

H. = High, Mod. = Moderate, L. = Low, Agree. = Extent of across-rater agreement in classifying the dialogue instance as involving self-disclosure. Intim. = Rater assessment of the level of intimacy in the dialogue instance.

**Fig 7:**
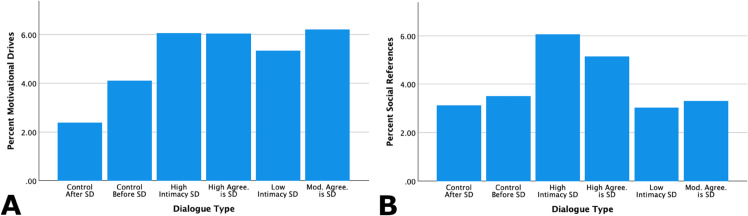
Further linguistic profiles of self-disclosure in conversational context: Motivational drives and social references. “Control before” and “control after” utterances are included in the leftmost two bars, high self-disclosure utterances are in the middle two bars, and low to moderate self-disclosure are in the rightmost two bars. A: Language related to motivational drives is higher in rater-agreed instances of self-disclosure than in the control dialogue segments. B: Social references are highest in both high intimacy instances of self-disclosure and high rater agreement. Note: Values on the y axes differ between plots. SD = self-disclosure, Mod. = Moderate, Agree. = Extent of across-rater agreement in classifying the dialogue instance as involving self-disclosure.

**Motivational drives and social references.** Language related to both motivational drives and social references was also relevant to instances of self-disclosure. Analysis shows that compared with the control CA-dialog samples, language related to motivational drives was elevated in instances evaluated as involving self-disclosure. Social references were similarly elevated during self-disclosure, particularly so for instances judged as involving a high degree of self-disclosure. Table 6 and Fig 7 show these trends.

**Table 6:** Linguistic characteristics of self-disclosure in ongoing conversational context: Motivational drives and social references.

LIWC Variable	Self-Disclosure Dialogue				Control Dialogue	
	H. agree.	Mod. agree.	H. intim.	L. intim.	Before	After
Social references	5.15	3.31	6.06	3.03	3.51	3.12
Motivational drives	6.05	6.20	6.06	5.33	4.11	2.39

Note: H. = High, Mod. = Moderate, L. = Low, Agree. = Extent of across-rater agreement in classifying the dialogue instance as involving self-disclosure. Intim. = Rater assessment of the level of intimacy in the dialogue instance.

**Positive and negative emotion.** We last evaluated separate counts for words reflecting positive vs. negative emotion to corroborate the findings for the summary LIWC measure of (positive) tone. The trends for positive and negative emotions were, as expected, in line with the findings for the summary measure. Specifically, words relating to positive affect were somewhat less frequent in the dialogue involving self-disclosure than in the control dialogue (the dialogue turns from the same speaker that occurred immediately before and after the self-disclosure). Table 7 demonstrates this analysis; as this is a corroboration of the Tone summary variable, a visual representation of these trends can be seen in Panel D of Fig 5.

**Table 7:** Linguistic characteristics of self-disclosure in ongoing conversational context: Positive and negative emotions.

LIWC Variable	Self-Disclosure Dialogue				Control Dialogue	
	H. agree.	Mod. agree.	H. intim.	L. intim.	Before	After
Positive emotions	3.07	3.10	1.99	4.01	3.34	4.36
Negative emotions	2.22	1.50	2.49	1.61	0.86	0.42

Note: H. = High, Mod. = Moderate, L. = Low, Agree. = Extent of across-rater agreement in classifying the dialogue instance as involving self-disclosure. Intim. = Rater assessment of the level of intimacy in the dialogue instance.

Several qualitative patterns are observed in the analysis of cognitive processes and personal pronouns, as well as the analysis of positive and negative emotions. It appears that, compared with the two types of control dialogue, instances involving self-disclosure are characterized by more frequent use of the personal pronoun ‘I’, some elevation in the frequency of words referring to cognitive processes, generally fewer words referring to positive emotion and more words referring to negative emotion, and a higher frequency of references to motivational drives. Compared with the two types of control dialogue, references to social processes seem to be selectively elevated for instances judged as involving a high degree of self-disclosure and that elicited high rates of classification as self-disclosure.

#### Relationships between dimensions of self-disclosure.

In **RQ3**, we asked how the identified dimensions of meaning for self-disclosure interrelated with one another. Analysis of the Phase II ratings from the 7-item survey revealed several important patterns in correlational relationships between the dimensions of self-disclosure that are aligned with previous literature. Notably, there was a significant positive correlation between rater agreement as to whether an instance was self-disclosure (that is, the detectability of self-disclosure) and the rated degree of intimacy of the self-disclosure (r = .42, p < .001). Intimacy was also associated with several other dimensions; there was a moderately negative correlation between the degree of self-disclosure and the valence of what is disclosed, such that instances rated as more intimate were also evaluated as involving more negative emotion (r = –.33, p < .001). There was also a small negative correlation between the degree of intimacy of the self-disclosure and the relatedness of the disclosure to the prior speaker’s prompt (r = –.15, p = .041).

Analysis of the evaluations concerning the content and mental verbs of responses were also revelatory. Highly agreed-upon instances of self-disclosure were moderately positively correlated with both the number of different categories of content (topics) (r = .28, p < .001) and the number of different categories of mental verbs (r = .31, p <.001) to which participants classified the instances as belonging. The mean number of topics indicated across all raters for these instances was 3.19 (95% CI [2.93, 3.45]). The mean number of different mental verbs indicated across all raters was 4.26 (95% CI [4.01, 4.50]). The degree of intimacy also interrelates with the number of different topics indicated; there was a significant positive correlation between the number of topics indicated by raters and the degree of intimacy of the self-disclosure (r = .33, p < .001). There was a very small positive relationship between the degree of intimacy and the number of different mental verbs indicated by raters (r = .11, p < .13).

Table 8 provides rater-based assessments for three different representative instances of self-disclosure, including their topic and the mental verb through which they were expressed. The four most common rater-based assessments of the content of the self-disclosure instances were (in order of frequency, across all stimuli): work/study, eating/sleeping, leisure, and health. For these rater-based assessments of the content of self-disclosure, approximately 55% were to the work/study content category, 14% were designated as about eating/sleeping, 11% as involving leisure, and 5% as involving health-related matters. The most common rater-based assessments for the mental verb of the self-disclosure were (in order of frequency, across all stimuli): feeling/emotional state, planning, factual information, personal preference, and thoughts/beliefs. For these rater-based assessments of the mental verb of self-disclosure, approximately one third (34%) were designated as involving affect; that is, feeling/emotional state, followed by planning (22%), factual statements (18%), personal preference (10%) and thoughts/beliefs (10%).

**Table 8:** Sample stimulus pairs and ratings.

Stimulus Pair	Content	Mental Verb
Speaker 1: How did it [work] go?		
Speaker 2: It went well. I like my job.	Work/study (94% agreement)	Personal preference (79% agreement)
Speaker 1: After the project meeting, you planned to call a friend at around five. How did it go?		
Speaker 2: It went really well. I called my friend; her name is [FRIEND NAME]...and we talked for thirty minutes.	Friendship (97% agreement)	Factual (56% agreement)
Speaker 1: After lunch, you planned to research grad school for about two hours. How did your research go?		
Speaker 2: < opens mouth> umm, it went okay. It was very stressful, so I had to stop and watch tv for a while.	Work/study (88% agreement)	Feeling/ emotional state (94% agreement)

Note: Percent agreement for these samples is calculated based on n = 32-36 raters.

There were no significant correlations between rater-based assessments of the valence of the self-disclosure and the number of different topics or referenced mental verbs of expression indicated by raters.

#### In-depth examination of rater-based inferences from instances of self-disclosure.

After completion of data collection in Phase II, three expert raters performed a preliminary analysis of the free-response rater inferences relating to the 176-item ‘Rating’ self-disclosure subset. This analysis revealed inferences about a speaker to be an exceptionally rich dimension of meaning both taken separately and together with other dimensions as part of our investigation into **RQ3**. Across all Phase II naive raters, each individual instance of self-disclosure was found to elicit as many as 20 or 30 different inferences, the majority of which were agreed upon by the experts as reasonable inferences from the self-disclosure instance. The inferences provided by naive raters also ranged from ones that were explicitly evident from the self-disclosure statement (i.e., a restating of what the speaker said), to indirectly evident from the statement, to deeper inferences that would require raters to draw upon preexisting background or social knowledge. Table 9 contains representative samples of the rater inferences drawn from instances of self-disclosure to illustrate the richness of these data.

**Table 9:** Samples of rater-provided inferences drawn from self-disclosure.

Stimulus Pair	Rater Inference
Speaker 1: After your test you planned to have a short snack at two thirty p.m. How was your snack?	They had a test
Speaker 2: Bigger than I thought. Because I was angry about the test.	They are in school They lack control over eating and emotions (disordered eating)
Speaker 1: When do you think you felt the most stress today?	They can bake
Speaker 2: ...probably between making the cake and...talking to you because I lost track of time a little bit.	(They are) unorganized Time management is important to them

Note: Rater inferences refer to the statement by the second speaker; ‘they’ refers to the second speaker.

#### Characterization of inferences.

Although many of the generated inferences were very similar to one another (e.g., “They enjoy cooking” vs. “They like to cook”), raters generated a very wide range of inferences. A number of inferences were unique or nearly unique, being generated by only one or two of the participants. (See Table 10 for examples.) To begin to more precisely quantify the number and variety of different inferences for each of the 176 stimuli, all inferences for a given stimulus were read and classified by an expert human rater (LF). Inferences that were evaluated as essentially identical or sufficiently similar to one another in meaning were grouped or classified together in the same “inference category.” Inferences that were evaluated as different in sense, meaning, or implication from one another were grouped into separate inference categories.

**Table 10:** Example categorization of inferences for one stimulus pair.

Stimulus Pair: *Speaker 1: Is there anything else I should know about baking?* *Speaker 2: Uh I’m making very delicious sweet rolls. Or sticky buns, sorry.*			
Inference	Judgment	Category	Size of Category
They bake a lot	Distinct	*A*	4
They are a baker	Distinct	*B*	4
They like sticky buns	Distinct	*C*	3
They like the rolls	Similar to *C*, merge	*C*	**4**
They might play Skyrim	Distinct	*D*	1

Note: Rater inferences refer to the statement by the second speaker; ‘they’ refers to the second speaker.

Across all 176 stimuli, this categorization process yielded a total of 3594 inference categories. The average number of categories per stimulus was 20.5, with each category per stimulus including a minimum of 1 and a maximum of 18 inferences. Given that this evaluation process was performed by only one rater, to provide convergent evidence for the validity and reliability of the categorization, semantic similarity scores within and between categories were calculated by examining cosine similarity scores, generated using embeddings from the all-MiniLM-L6-v2 transformer hosted on Hugging Face, a major repository for models and data used for machine learning applications [74]. These analyses provided strong support for greater similarity of meanings for the inferences grouped within categories. Specifically, the mean similarity score for inferences within the same categories was .72 (median = .71, SD = .07), whereas the mean similarity score for inferences assigned to different categories was .35 (median = .33, SD = .07).

A full analysis of the naive raters’ inferences drawn from self-disclosure will be reserved for future work.

#### Exploratory analysis: Further examination of the effects of the Wizard of Oz protocol.

In Phase I, we performed some exploratory analyses regarding the success of the Wizard of Oz protocol, and whether observed participant behaviors were attributable to participants suspecting they were speaking with a human operator. In Phase II, we expanded this analysis beyond the amount of self-disclosure that occurred and examined rater evaluations of instances of self-disclosure. In this exploratory analysis, we linked Phase I and Phase II by investigating what impact, if any, Phase I participants’ beliefs regarding whether or not they were speaking to a human had on the ratings provided by participants in Phase II. In other words, if the participant interacting with the WoZ system believed they were speaking to an artificial agent, how uniformly were their extracted dialogue snippets rated by participants in Phase II? Is there a measurable difference between how these dialogue snippets were rated versus dialogue snippets from participants who suspected they were speaking to a human?

To answer these questions, we divided our Phase I participants and stimuli into three groups: stimuli extracted from dialogues from all Phase I participants (n = 176 stimulus pairs, N = 27 participants), stimuli from naïve participants only (n = 55 stimulus pairs, N = 13 participants) and stimuli drawn from the first experimental session as a proxy for all participants being naïve (n = 53 stimulus pairs, N = 27 participants). For each grouping, we then analyzed the ratings provided by participants in Phase II.

In the results presented in the discussion of relations between dimensions of self-disclosure, we saw evidence that the dimension of intimacy was perhaps one of the strongest indicators of self-disclosure. In line with these results, analysis of ratings of intimacy across these three groups of Phase I participants showed markedly similar patterns. The average ratings of intimacy when raters positively identified a stimulus pair as containing self-disclosure were consistent across all three groups (2.64, 2.40, and 2.47 respectively), as were average ratings of intimacy when raters indicated a stimulus pair only possibly contained self-disclosure (2.10, 1.85, and 1.71 respectively). Most telling was perhaps the similarity in *gaps* between the average intimacy levels for ‘yes’ and ‘possibly’ responses, including a nearly identical gap between stimuli drawn from all participants vs. stimuli drawn from naïve participants only (0.54, 0.55, and 0.76 respectively). The full results from this analysis, including both dimensions of meaning from the proposed ontology and psycholinguistic characteristics from LIWC, are presented in Appendix: Naive participants.

## Discussion

### Key findings

This two-phase study sought to answer three primary research questions. In Phase I, we explored **RQ1** and its subquestions regarding the characteristics of dialogue in interactions with conversational systems where a user participated in self-disclosure. **RQ1** itself asked whether self-disclosure actually occurred in user dialogues with a task-oriented dialogue system, and we found strong evidence that users do participate in varying amounts of self-disclosure in these interactions. As part of our investigation, we asked about sociolinguistic features of these dialogues as a whole. Our results here strongly aligned us with existing self-disclosure literature. We found that dialogues in which self-disclosure occurred were associated with higher word count in general. We also saw that dialogues with self-disclosure, when compared to dialogues without self-disclosure, contained higher levels of Authenticity as measured by LIWC, lower levels of Analytical Language, and higher word counts related to motivational drives and “I” statements.

In Phase II, we applied our newly-developed ontology of self-disclosure to the conversational data collected in Phase I. In **RQ2**, we sought to characterize individual instances of self-disclosure using the same psycholinguistic measures as we used in **RQ1**. We found further evidence supporting both the selection of candidate instances of self-disclosure and the rating scale itself. Analysis of the individual instances displayed many of the same characteristics as the trends seen in the holistic dialogues. Candidate instances that raters strongly agreed contained self-disclosure had higher levels of the use of the “I” pronoun, as well as language related to motivational drives and social processes.

The main thrust of our investigation in Phase II was our third research question. We asked about the interplay between identified dimensions of self-disclosure in **RQ3**. The strongest relationships we found once again situated our ontology well within the literature; instances of self-disclosure that were higher in intimacy had a moderate tendency to be more negatively valenced. We also found a small negative correlation between intimacy of self-disclosure and the relatedness to the previous turns in conversation. Intimacy was further associated with a greater diversity of both rater-identified topic and mental verb. As a related question, we also explored corollaries with **RQ2** and examined the relationship between strength of rater agreement and the identified dimensions in the ontology. Our strongest evidence was once again along the dimension of intimacy – the highest inter-rater agreement as to whether a dialogue snippet contained self-disclosure occurred in instances that were rated as highly intimate. In parallel to the results in both **RQ1** and **RQ2**, inter-rater agreement was slightly stronger in instances with lower tone or more negative emotional content.

### Practical implications

The results of the work presented here provide an initial picture of user-disclosing behaviors in interactions with a task-oriented agent that has minimal social capabilities. The core of our findings – that diverse self-disclosure does regularly occur with these systems regardless of the absence of social cues that might imply any form of reciprocity – leads to several important design implications for future task-oriented systems, particularly those meant for long-term deployment.

#### The importance of social reasoning.

Our work suggests that self-disclosure may occur with any type of dialogue system, regardless of social capability. Analyzing these instances of self-disclosure further reveals that what is disclosed covers a wide array of any number of dimensions, especially topics and mental verbs. Thus, it is apparent that users will provide a wealth of information naturally and without prompting – information that is yet untapped when it comes to user customization and personalization. Taken together, these facts highlight the necessity of socially-grounded commonsense reasoning for conversational systems, even if a given system is not intended to be used for strictly social or more open domain interactions. Self-disclosure is a social phenomenon, and the information contained is so broad that reasoning with its contents may require a more sophisticated model than designers might otherwise select for a task-oriented system. This work provides additional support to the ongoing argument in the field that more well-rounded systems – that is, systems trained for multiple domains rather than narrow task-relevant subsets – exhibit better behavior overall, precisely because so much information is conveyed between the lines (see, e.g., [75–77]).

#### Dimensions of meaning and agent design.

The dimensions of self-disclosure included as part of this ontology further highlight the importance of socially-grounded commonsense reasoning regardless of intended use case. Each dimension lends itself to different aspects of agent design, from conversational flow to knowledge organization. While the potential uses of some dimensions, such as topic, are self-explanatory, the new dimensions proposed in this ontology may be particularly salient to behavioral planning for agents. Separating the mental verb of self-disclosure from its topic is especially promising for this purpose. Table 2 demonstrates that the same topic may occur in very diverse ways throughout the course of a conversation, at which point the topic itself becomes more or less salient depending on the mental verb. This may be even more important for a task-oriented agent enabled with some degree of proactivity. For example, the mental verb of habit (‘I make beef stew once a week’) may prompt an agent to add stew beef to a weekly shopping list; the mental verb of thought/belief or judgment (‘The store seems to have the best markdowns on stew beef on Mondays’) may add or move a weekly shopping reminder to the calendar on Monday. On the other hand, the mental verb of preference (‘I like short ribs better than sirloin in beef stew’) may prompt an agent to update the shopping list or add a weekly search for sales on different cuts of beef to its standard behaviors. This variety also demonstrates that the ways in which these mental verbs dictate behaviors, thereby directly or indirectly exposing the user model to the user, is another very open and information-rich avenue of investigation worth exploring.

The dimension of conversational context is also worth further exploration. As a dimension in the proposed ontology of self-disclosure, it serves as a measure of how much a discloser is departing from the immediately previous conversation during the turn containing self-disclosure. In applying the ontology, this is captured as a Likert scale rating of the degree of relatedness between the utterance containing self-disclosure and the previous turn from a different speaker. In the current study, we also examined the linguistic profile of disclosure in conversational context by capturing psycholinguistic characteristics of utterances of self-disclosure as well as the turns immediately preceding and immediately following the disclosure. This allows us to capture a psycholinguistic ‘fingerprint’ of self-disclosure in addition to a rating of how much the conversation is branching at the point of disclosure. One of the primary uses of these two meanings of context (Likert scale rating or psycholinguistic profile), taken separately or together, may be as a cue that self-disclosure is more likely, and thus an agent should ‘pay more attention’ to points at which a user departs from the current context, or points at which a user utterance has a linguistic footprint separate from previous turns in the conversation. In the case of a task-oriented system the psycholinguistic profile may be most relevant, and may simply mean that a given turn by a user is flagged for potential use in a user model; in a system with more social capability, the full ontological dimension of context as a measure of relatedness to other turns may be more relevant. In an explicitly social system, a departure from context designates a potential branching point in the conversation, analogous to a bookmark. A natural behavior at such a point might be to diverge from the conversation to probe whatever is disclosed further, before returning to the prior topic of conversation.

#### Building personalized user models.

One of the challenges that has long plagued task-oriented conversational systems is the failure of users to adopt systems for long-term use [27, 29, 49]. Thus, ways to overcome this hurdle are of primary interest to the field [32]. We have previously noted that there is a wide variety of information disclosed by users – understanding what is disclosed is one thing, but effectively and appropriately using it is another. All of this information can be used to create a more tailored and adaptable user experience, which may be particularly important for encouraging adoption of more task-oriented systems [9, 25, 78]. For example, a user whose dialogue with a system reveals a dislike of cold weather may appreciate a reminder to wear a jacket, or a user who has previously disclosed that they park on the street may benefit from a system that can add a ‘check for snow emergency announcements’ reminder to a to-do list during the winter. The incredible variety of information available also points to a related key observation, however – being able to extract information about a user does not necessarily mean it *should* be used. This also suggests a potentially rich avenue of investigation for further work.

The question of what user information to use and when means that personalization also includes maintaining a mental model of users. Social reasoning and developing interpersonal relationships requires creating mental models of others and understanding that each person maintains different internal states [60, 61]. The proposed ontology of self-disclosure includes the dimension of mental verb for this reason. Much of the potential internal state of users can be directly or indirectly expressed through acts of self-disclosure, particularly when examining mental verbs and the relationships between dimensions of self-disclosure. Maintaining and reasoning with knowledge about the internal state of a user mirrors important social processes that naturally occur in interpersonal relationships. One such process is perspective taking, or ‘walking a mile in someone else’s shoes,’ which is a core component of empathy, building rapport, and goal alignment. Including mental verbs – preferences, habits, emotional states, etc. – in a user model may allow an agent to roughly approximate perspective taking and respond appropriately to the detected internal state of a user.

#### Interactivity and disclosure over time.

Since our findings clearly suggest that understanding and reasoning with information disclosed by users is important regardless of the intended use of a system, we can also anticipate further design considerations, particularly as applies to systems meant for long-term use. In line with building more personal knowledge of users through what is disclosed, it may also be important to explicitly maintain interaction history with users to a much greater extent than commercially available systems do today. As previously noted, from within the CASA theoretical framework, intelligent systems, particularly those that are language-based (and perhaps especially so for those that are also voice-based), inherently tend to send (or imply) social cues regardless of other aspects of system design. This also leads users to approach these systems as highly interactive – that is, users may approach language-based systems as things that will support interactions over time and that also possess some knowledge of their prior interactions with the system (their interaction history) [32, 41]. This somewhat subconscious (mis)attribution by users may also be a cause of failing to meet user expectations, which further discourages long-term use [27, 29, 30, 35]. Systems designed to understand and reason with self-disclosure should therefore be capable of understanding some user context and history.

Our results also support that maintaining some knowledge of history is important in continuing to encourage interaction with a conversational system. While the current study is not as longitudinal as others, our results align very well with previous work that demonstrates self-disclosure can occur with conversational systems over much longer periods of time [48, 79]. As noted previously, recent work proposes a model of relationship development with conversational systems in which there is not much expectation of reciprocity from the system, so, over the longer term self-disclosure is likely to continue to occur regardless of the presence or absence of reciprocity [48]. This model also suggests that – even in the absence of actual or expected reciprocity – relationships with conversational systems can stabilize over time rather than stagnate, meaning again that self-disclosure can be expected to continue to occur in future interactions.

#### User privacy and data security.

Our findings indicating diverse disclosure and continued disclosure over time also suggest that we cannot fully predict the where, when, and what of disclosure, which leads to concerns regarding the protection of user data. This is exacerbated by the idea that self-disclosure, particularly more intimate self-disclosure regarding highly sensitive topics, is perhaps more a longitudinally manifested phenomenon than anything else [10]. Current models such as Skjuve’s three-phase relationship building model indicate that self-disclosure occurs with conversational systems over the long term [48]. Combined with the idea that self-disclosure becomes more intimate over time, we may expect to see more sensitive information being disclosed by users of systems in long-term deployment. Future task-oriented systems meant to encourage long-term use should therefore treat all user data as sensitive and maintain control over these data as much as possible. This might include measures such as treating all data in a user model as potentially identifying health data, and ensuring that any user models and all conversational data are password protected, only stored locally, cannot be directly queried by any user, and can be completely reset at any time.

Further transparency about data protection and security measures may also help reassure users and encourage more long-term use. Many conversational agents may come in the form of dialogue systems attached to either a personal device like a watch or phone or to something less mobile, like a smart home system. In these cases, promoting long-term use and consistent levels of openness may mean designing for others in the area as well. A personalized smart device in these cases might be passphrase, gesture, or voice print locked in order to prevent user’s personal information inadvertently being exposed to others. In the WoZ setup used to collect conversational data in this study, users were alone in the room with the ‘prototype’ system except for sometimes one research team member, and so the only parties to their conversations were the users themselves, the researcher in the room if present, and the researcher using the soundboard. This is significantly harder to enforce in, for example, a family environment, so for maximum comfort and privacy users should also be able to customize and fine tune the numbers and types of security measures they wish to have in place.

#### Practical application of the ontology.

This work has highlighted the ubiquitousness of self-disclosure, as well as its complexity. It should also be stressed that a multi-dimensional understanding of self-disclosure such as we present in this work may be very valuable for multiple aspects of CA and interaction design, regardless of how socially-oriented a CA is intended to be. Medium- and longer-term work in this area should take a practical approach and explore the real-time applications of the ontology in interactions with users, particularly as a cue for behavioral and response adaptation. One potential application of the ontology is as a selection mechanism for active listening techniques. A conversational partner’s reactions to and interactions with what is being disclosed have a wide-ranging impact on both the immediate conversation and the relationship as a whole [80, 81]. The use of active listening, in which a listener uses verbal and nonverbal cues to signal to a speaker that they are receptive and engaged, is valuable in maintaining relationships and promoting further disclosure [82]. Evidence suggests this is true for CA listeners as well as humans [83, 84]. At any given time, one active listening technique may be more appropriate or well-received than another. Prompting questions may be significantly less well-received when someone is recounting a negative memory than discussing exercise preferences, for example. Ontological dimensions such as mental verb, intimacy, or emotional valence may be useful in guiding listener strategy selection and increasing user satisfaction.

A second, related potential application of the ontology is in guiding agent initiative in interactions. Selective or mixed initiative taking is a difficult problem [85, 86]. A user discussing meal planning might be appreciative of a CA volunteering to set up a weekly recurring search of store websites for the best produce deals. A user lamenting that their date tonight was a disaster might find it off-putting if a CA asked whether it should be made a recurring event. A CA that is able to classify disclosure using ontological dimensions like topic and mental verb may be able to better select when to take initiative, which in turn would lead to increased user satisfaction and longer-term adoption.

### Limitations and future directions

The current work provides a launching point for many further questions regarding the use of self-disclosure with task-oriented systems. The most salient limitation of our work is that while our data comes from multiple sessions of user interaction with the prototype over a period of days, an even longer study would allow us to develop a more detailed and extensive picture of disclosing behaviors with task-oriented systems. The trends noted in the LIWC summary variables, such as authenticity and clout, indicate our data does extend beyond the initial novelty/exploration phase of interacting with new technology and points toward our findings being applicable over the longer term. This is a core open question for future exploration. Another limitation of our findings is that the experiments were carried out only with younger adults who typically had comparatively routine/structured daily schedules. Our current and future work extends this experimental paradigm to older adults with more varied schedules [87], and we will also be examining the variety and extent of self-disclosure in this age group in the hopes of contributing to more robust characterization and understanding of the behavioral differences between very distinct groups of potential users of CAs. It is also important to note that certain dimensions and characteristics of self-disclosure, namely valence and the LIWC measure of emotional tone, may have been influenced by specific aspects of the experimental structure. Since the original interactions with the “prototype” CA had a focus on daily and event-related stress levels, negative emotional valence may have been somewhat increased, and positive tone somewhat decreased, in comparison with the levels that might be seen in instances of self-disclosure emerging during more general social interactions with the prototype. The possible contribution of this aspect of the protocol is also being addressed in our ongoing work, in which the CA stress-related prompts have been removed from the interaction design.

We also note that the questions investigated in this study are related to the structuring and size of the available experimental data. All data used for the practical application of the ontology in Phase II was generated by participants conversing with the ‘prototype’ in Phase I. While we had a reasonable number of interactions from Phase I (N = 114) with which we could perform linguistic analysis and investigate macro-level trends related to dimensions of the ontology, we had a relatively small number of participants in the same phase (N = 33). The smaller number of participants was largely due to the intensity of the data collection process in Phase I and the sample was predominantly female. However, this leaves open some very pertinent questions about between-person differences in user behavior when it comes to self-disclosing. Further studies with a larger and more demographically balanced participant pool may address questions of the effects of user characteristics, such as gender or age, on disclosing behaviors with CAs.

In addition to expanding participant diversity and modifying the interaction design, much of our future work will focus on in-depth study of the rater-provided inferences drawn from instances of self-disclosure. The preliminary results discussed previously and briefly illustrated in Tables 9 and 10 display an extraordinary richness of information worthy of separate investigation. In the immediate future, we will be fully categorizing and analyzing rater inferences for every instance of self-disclosure.

## Conclusion

In this two-phase study, we have shown a profile of user-disclosing behavior with a minimally social CA such as a personal voice-based digital assistant. Our findings demonstrate that users disclose pertinent personal information to such a conversational system even in the absence of the majority of social cues and reciprocity. Our seven-point assessment of the multidimensional nature of any instance of self-disclosure provides a strong grounding for future research of self-disclosure that incorporates not only the content or depth of self-disclosure, but additional important dimensions such as the mental verb underpinning the disclosure and the rich set of speaker characteristics that can be inferred from any given instance of self-disclosure. We have also demonstrated methodological convergence between sociolinguistic measures of language use and rater-based assessments of self-disclosure. The work presented here underscores and supports the view of self-disclosure as a highly complex, multifaceted social phenomenon worthy of studying in the context of conversational agent design and development. This work also demonstrates that humans are capable raters in the task of explicitly analyzing self-disclosure across multiple dimensions of meaning, indicating that using this ontology to annotate instances of self-disclosure in conversation may help provide a novel and more comprehensive framework for a multifaceted analysis of self-disclosure for both humans and intelligent agents.

## Supporting information

Appendix: Naive participants Effects of WoZ protocol on participant disclosing behavior. Table 11 presents the full analysis of representative characteristics of disclosure for three participant subgroups, including both dimensions of meaning from the proposed ontology and psycholinguistic characteristics as defined by LIWC.
(PDF)
