# Supplementary material for: Personally speaking: Developing and evaluating an ontology of dimensions of meaning for self-disclosure with a conversational assistant
Source: PLoS One. 2026 Feb 20;21(2):e0341640. doi: 10.1371/journal.pone.0341640 (PMC12923126; doi:10.1371/journal.pone.0341640)
Supplement: Appendix: Naive participants — Table 11 presents the full analysis of representative characteristics of disclosure for three participant subgroups, including both dimensions of meaning from the proposed ontology and psycholinguistic characteristics as defined by LIWC. (PDF) [file pone.0341640.s001.pdf]

Supporting information

Appendix: Naive participants. Effects of WoZ protocol on participant disclosing behavior. Table 5 presents the full analysis of representative characteristics of disclosure for three participant subgroups, including both dimensions of meaning from the proposed ontology and psycholinguistic characteristics as defined by LIWC.

Table 5. Analysis of Representative Characteristics of Disclosure from Participant Groups

Measure	All sessions All participants (N = 27[a], n instances = 176)			All sessions Naive participants only (N = 13, n instances = 55)			Session 1 only All participants (N = 27, n instances = 53)		
**Self-disclosure?**	**Yes**	**Poss.**	**No**	**Yes**	**Poss.**	**No**	**Yes**	**Poss.**	**No**
Average intimacy	2.64	2.10	–	2.40	1.85	–	2.47	1.71	–
Average valence	2.95	2.86	–	2.85	2.94	–	2.94	3.14	–
LIWC Avg. WC	130			154			74.68		
LIWC Avg. Anal.	23.12			31.56			21.03		
LIWC Avg. Cl.	7.15			5.77			4.19		
LIWC Avg. Auth.	82.62			80.19			74.20		
LIWC Avg. Tone	43.48			39.66			41.04		

[a]N = 27 for all participants whose agreed-upon potential instances of self-disclosure were rated in Phase II. LIWC summary variables abbreviated for space; WC = Word Count, Anal. = Analytic, Cl. = Clout, Auth. = Authenticity.

.
